# Energy Transfer Efficiency from ZnO-Nanocrystals to Eu^3+^ Ions Embedded in SiO_2_ Film for Emission at 614 nm

**DOI:** 10.3390/ma10080930

**Published:** 2017-08-10

**Authors:** Vivek Mangalam, Kantisara Pita

**Affiliations:** 1 OPTIMUS, Centre for OptoElectronics and Biophotonics, School of Electrical and Electronic Engineering, Nanyang Technological University (NTU), Block S2, 50 Nanyang Avenue, Singapore 639798, Singapore; vivek014@e.ntu.edu.sg; 2 CINTRA, CNRS-NTU-Thales UMI 3288, Research Techno Plaza, 50 Nanyang Drive, Border X Block, Level 6, Singapore 637553, Singapore

**Keywords:** zinc oxide nanocrystals, energy transfer efficiency, Europium(III) ions, time-resolved photoluminescence

## Abstract

In this work, we study the energy transfer mechanism from ZnO nanocrystals (ZnO-nc) to Eu^3+^ ions by fabricating thin-film samples of ZnO-nc and Eu^3+^ ions embedded in a SiO_2_ matrix using the low-cost sol-gel technique. The time-resolved photoluminescence (TRPL) measurements from the samples were analyzed to understand the contribution of energy transfer from the various ZnO-nc emission centers to Eu^3+^ ions. The decay time obtained from the TRPL measurements was used to calculate the energy transfer efficiencies from the ZnO-nc emission centers, and these results were compared with the energy transfer efficiencies calculated from steady-state photoluminescence emission results. The results in this work show that high transfer efficiencies from the excitonic and Zn defect emission centers is mostly due to the energy transfer from ZnO-nc to Eu^3+^ ions which results in the radiative emission from the Eu^3+^ ions at 614 nm, while the energy transfer from the oxygen defect emissions is most probably due to the energy transfer from ZnO-nc to the new defects created due to the incorporation of the Eu^3+^ ions.

## 1. Introduction

The study of the excitation of rare-earth (RE) ions through energy transfers from semiconductor nanocrystals, especially from Zinc oxide nanocrystals (ZnO-nc) has attracted a lot of attention lately. The emission from the 3.37 eV wide bandgap of ZnO-nc has been shown to efficiently excite a wide variety of RE ions ranging from Ce^3+^ to Yb^3+^ ions [1,2,3,4,5,6,7,8]. Nonetheless, the mechanism of energy transfer from ZnO-nc to RE ions is not well understood. For instance, Huang et al. [8] report that the energy transfer from ZnO-nc to RE ions, specifically Eu^3+^ ions, is mainly due to the defects in the ZnO-nc. However, reports by Luo et al. [5] show that the energy transfer from ZnO-nc could be due to a combination of the band edge emission from the ZnO-nc and the defects in the ZnO-nc. Furthermore, in most of these works, the study of the energy transfer from ZnO-nc is based on steady-state photoluminescence (PL) emission from the nanocrystals, while there have been fewer studies based on time-resolved photoluminescence (TRPL) measurements from the ZnO-nc [2,3]. Moreover, there have been almost no studies that aim to further the understanding of the de3cay dynamics of various ZnO-nc emission centers and their effect on the efficiency of the energy transfer mechanism from ZnO-nc to RE ions. This article is a direct continuation of our earlier work [9], in which the steady-state PL emission from ZnO-nc was studied to elucidate the contribution of seven ZnO-nc emission centers in the energy transfer process to Eu^3+^ ions. These seven emission centers of the ZnO-nc in SiO_2_, centered at 360, 378, 396, 417, 450, 500, and 575 nm [10], were identified as band-edge emissions from the smallest ZnO-nc, which possibly experiences some quantum confinement effect (QC) [11], excitonic emission (EE) [12,13], and five different defect state emissions, namely Zn defect (Zn_i_ to V_Zn_) [14], oxygen interstitial defect (O_i_) [15,16], and oxygen vacancy defects (V_o_, VȮ, VӦ) [14,17,18], respectively. In this work, we study the contribution of energy transfer from various ZnO-nc emission centers to Eu^3+^ ions based on TRPL measurements. We calculate the decay time from the TRPL measurements for various ZnO-nc emission centers and then calculate the efficiency of energy transfer from each of the ZnO-nc emission centers to the Eu^3+^ ions. Furthermore, the transfer efficiency is also calculated from steady-state PL emission data for comparison. The results in this work show high transfer efficiencies from the EE (at 378 nm) and Zn_i_ to V_Zn_ (at 396 nm) emission centers, mostly due to the energy transfer from ZnO-nc to Eu^3+^ ions which results in radiative emission from Eu^3+^ ions at 614 nm, while the energy transfer from the oxygen defect emissions at 417, 450, 500, and 575 nm is most probably due to the energy transfer from ZnO-nc to the new defects created due to the incorporation of the Eu^3+^ ions, as proposed in our previous work [9]. Understanding the energy transfer mechanism from ZnO-nc to Eu^3+^ ions will help to further the understanding of the energy transfer process to RE ions and in the development of energy efficient RE ion-based light sources such as lasers, amplifiers, phosphors, etc. that can be excited by the sensitizing effect of semiconductor nanocrystals.

## 2. Results and Discussion

In this work, we studied the TRPL spectra of two types of thin-film samples; namely ZnO-nc in SiO_2_ (ZnO-nc:SiO_2_) and ZnO-nc in SiO_2_ co-doped with 12 mol % Eu^3+^ ions (Eu^3+^:ZnO-nc:SiO_2_). The 12 mol % Eu^3+^:ZnO-nc:SiO_2_ sample was used in this study because the 12 mol % sample has the highest PL emission from the Eu^3+^ ions at 614 nm due to energy transfer from ZnO-nc, in comparison with 4, 8, and 16 mol % Eu^3+^:ZnO-nc:SiO_2_ samples, which has been reported in our previous work [9]. The TRPL spectra were measured at each of the peak wavelengths of the seven ZnO-nc emission centers. The TRPL signals from the last three longest wavelengths of the seven signals, namely at 450, 500 and 575 nm, were weak compared to the other four emissions at 360, 378, 396 and 417 nm.

**Figure 1:**
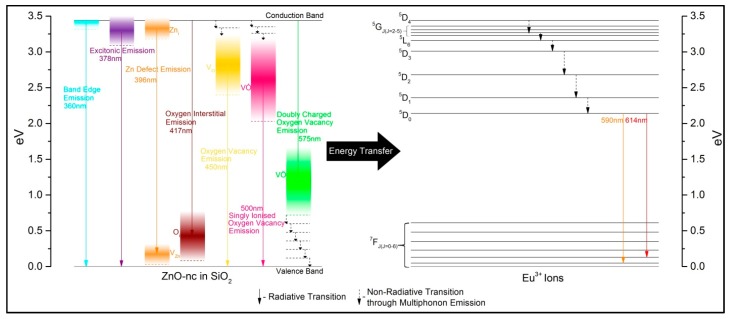
Energy band diagram showing the energy transfer from ZnO-nc to Eu^3+^ ions in SiO_2_ together with the various transitions corresponding to the emissions from ZnO-nc and Eu^3+^ ions.

In our PL measurements [9], we also observed that the 450, 500 and 575 nm emissions have the lowest emission intensity. These weak emissions are possibly due to the larger number of phonons required as compared to the emissions at 360, 378, 396 and 417 nm, shown schematically in Figure 1 (the energy band diagram of ZnO-nc in SiO_2_ showing various transitions). The number of phonon emissions required by the 450, 500 and 575 nm emissions is estimated to be around three to nine times more than those required by the 417 nm emission process. The probability of multi-phonon emissions decreases with increasing the number of phonons required for the de-excitation process, leading to a lower number of excited state ZnO-nc electrons being involved in the emissions at 450, 500 and 575 nm, causing weak emissions at these wavelengths. The energy band diagram in Figure 1 also illustrates the energy transfer from various ZnO-nc emission centers to Eu^3+^ ions in the SiO_2_ matrix, together with the various transitions corresponding to the emissions from ZnO nanocrystals and Eu^3+^ ions. This figure is a modified version of the band diagram reported in our earlier work [9], where the bandwidth of the various ZnO-nc emission centers is represented using the colour gradients in the broad vertical energy bands. Here, we note that there is a possibility of back energy transfer from the excited Eu^3+^ ions to the ZnO-nc, especially from the ^5^D_0_ state of Eu^3+^ to the VȮ defect center in the ZnO-nc with the cooperation of phonons. However, we do not consider the effect of the energy back transfer process on the PL and TRPL emissions of the ZnO-nc in this study.

### 2.1. Transfer Efficiency from Time-Resolved Photoluminescence Emission

**Figure 2:**
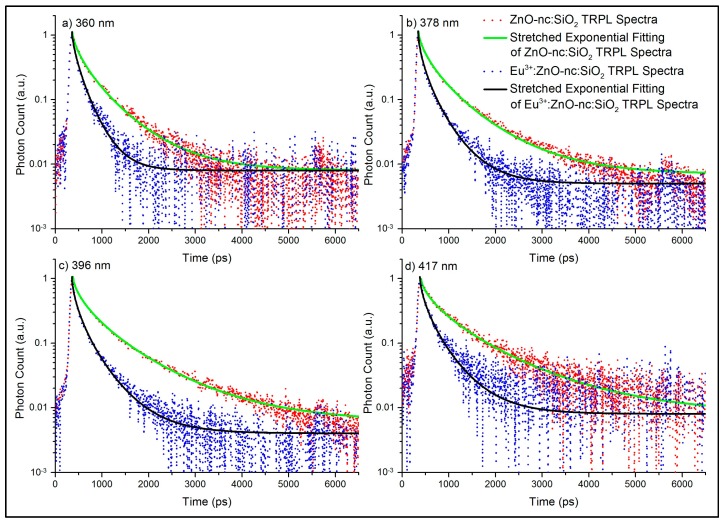
The time-resolved photoluminescence (TRPL) spectra of the ZnO-nc:SiO_2_ and Eu^3+^:ZnO-nc:SiO_2_ samples measured at (**a**) 360 nm; (**b**) 378 nm; (**c**) 396 nm; and (**d**) 417 nm, along with their respective stretched exponential fitting curves.

Figure 2 shows the normalized TRPL spectra (the decay of the normalized PL emission intensity with respect to time) of ZnO-nc:SiO_2_ and Eu^3+^:ZnO-nc:SiO_2_ samples at 360, 378, 396 and 417 nm. We can clearly see that the PL intensity of the sample with the Eu^3+^ ions decays faster than the sample without Eu^3+^ ions at all emission wavelengths. The decay of the PL emission in the ZnO-nc:SiO_2_ sample is due to the radiative and non-radiative de-excitation processes in the ZnO-nc. However, the decay of the PL emission in the Eu^3+^:ZnO-nc:SiO_2_ sample has additional components that contribute to the decay rate, namely (i) the energy transfer from ZnO-nc to Eu^3+^ ions which results in the radiative emission from Eu^3+^ ions at 614 nm, and (ii) the energy transfer from ZnO-nc to the additional new defects created due to the incorporation of the Eu^3+^ ions [9]. To study the contribution of the energy transfers from the ZnO-nc to the Eu^3+^ ions and the defects created by the Eu^3+^ ions, the intensities of the different TRPL spectra of the two samples are expressed using the stretched exponential decay fitting function [19] as follows:

(1) $$I\left(t\right)=I_{o}e^{-{\left(\frac{t}{\tau }\right)}^{\beta }}$$

where I(t) is the intensity of the TRPL measurement at time *t*, $I_{o}$ is the intensity of the TRPL measurement at t=0, τ is the decay time of the TRPL intensity, and *β* is the stretching exponential coefficient, which is a wavelength-dependent constant. The stretched exponential decay fitting function is the most widely used fitting function to represent the TRPL decay curves of ZnO-nc [19], and semiconductor nanocrystals in general [20,21,22,23], as they account for the complex nature of the decay curves which cannot be expressed using simple exponential decay functions. Using the stretch exponential decay fitting function, we obtained very good fitting curves for the TRPL spectra of the ZnO-nc. In the stretched exponential decay equation, *β* ranges from 0 to 1 and is a measure of interaction between identical ZnO-nc emission centers. This interaction, for instance, could be in the form of the migration of excitons between ZnO-nc or the migration of carriers between identical ZnO-nc defect centers [22,23]. The value of *β* approaches 0 when the effect of interaction between two or more identical ZnO-nc emission centers is large, and *β* approaches 1 when the effect of interaction between the identical emission centers is negligible [22,23]. For the 360-nm band edge emission (QC) of the ZnO-nc, the value of *β* is 0.65 for the sample without Eu^3+^ ions and 0.69 for the sample with Eu^3+^ ions, as shown in Table 1. However, for the 378-nm excitonic emission (EE), 396-nm Zn defect (Zn_i_ to V_Zn_) emission, and 417-nm oxygen interstitial defect (O_i_) emission, the *β* values are around 0.61–0.62 for the samples with and without Eu^3+^ ions. These results indicate that the 360-nm band edge emission (QC) experiences lesser interactions compared to the EE, Zn_i_ to V_Zn_, and O_i_ emissions. The weaker interaction of the 360-nm band edge emission (QC) center could be due to the smaller size of the ZnO-nc, as this emission is attributed to the possible quantum confinement effect. Table 1 also show the decay time τ for different ZnO-nc emission centers of the ZnO-nc:SiO_2_ and Eu^3+^:ZnO-nc:SiO_2_ samples. The decay time τ is primarily the lifetime of the electrons in the excited state of the ZnO-nc emission center, and its values depend on the rates of the different de-excitation processes from the ZnO-nc emission centers. For instance, the decay time of any ZnO-nc emission center in the ZnO-nc:SiO_2_ sample $\left(\tau^{ZnO-nc:SiO_{2}}\right)$ is given by:

(2) $$\tau^{ZnO-nc:SiO_{2}}=\frac{1}{R^{r}+R^{nr}}$$

where *R^r^* is the rate of radiative emission and *R^n^*^r^ is the rate of non-radiative emission from the ZnO-nc emission center. Similarly, the decay time of any ZnO-nc emission center in the Eu^3+^:ZnO-nc:SiO_2_ sample $\left(\tau^{Eu^{3+}:ZnO-nc:SiO_{2}}\right)$ is given by:

(3) $$\tau^{Eu^{3+}:ZnO-nc:SiO_{2}}=\frac{1}{R^{r}+R^{nr}+R^{tr}}$$

where *R^tr^* is the combined rate of energy transfer from ZnO-nc to Eu^3+^ ions which results in the radiative emission from the Eu^3+^ ions at 614 nm and energy transfer from ZnO-nc to the additional new defects created due to the incorporation of the Eu^3+^ ions. As expected, the decay time for the sample with Eu^3+^ ions ($\tau^{Eu^{3+}:ZnO-nc:SiO_{2}}$) is lower than the decay time for the sample without Eu^3+^ ions ($\tau^{ZnO-nc:SiO_{2}}$) for all wavelengths, due to the additional energy transfer decay mechanisms in the Eu^3+^:ZnO-nc:SiO_2_ sample.

From the decay time results obtained from the TRPL measurements in Table 1, the energy transfer efficiency, denoted by $E_{T}^{TRPL}$, was then calculated according to the following equation:

(4) $$\mathrm{Transfer}\;\mathrm{efficiency}\;\left(E_{T}^{TRPL}\right)=\frac{R^{tr}}{R^{r}+R^{nr}+R^{tr}}=1-\frac{\tau^{Eu^{3+}:ZnO-nc:SiO_{2}}}{\tau^{ZnO-nc:SiO_{2}}}$$

**Figure 3:**
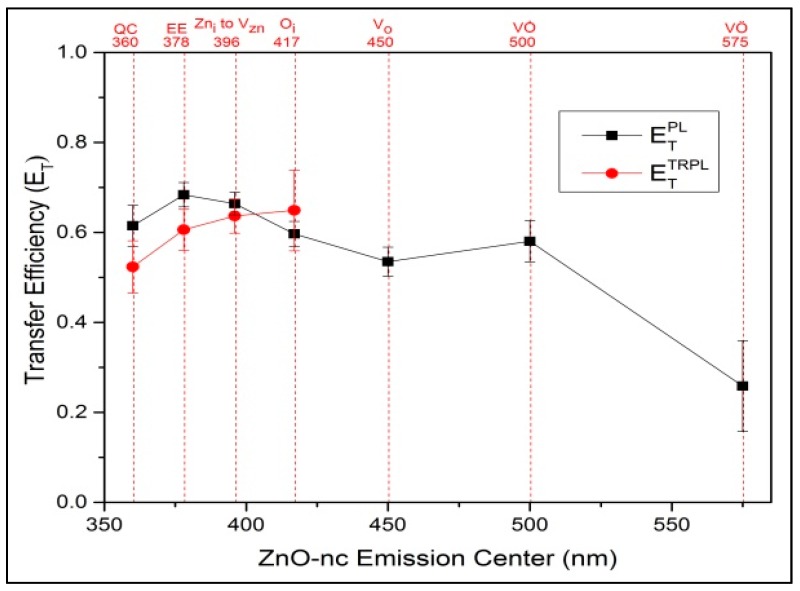
Transfer efficiency of ZnO nanocrystals emission centers due to the incorporation of the Eu^3+^ ions, calculated from the time-resolved photoluminescence (TRPL) spectra and steady-state photoluminescence (PL) emission data, along with their respective error bars.

The $E_{T}^{TRPL}$ transfer efficiency calculated for each of the four ZnO-nc emission centers is shown in Figure 3.

### 2.2. Transfer Efficiency from Steady-State Photoluminescence Emission

Furthermore, for comparison, the transfer efficiency of each of the seven ZnO-nc emission centers was also calculated from the steady-state PL intensity measurements of the two samples in this study, ZnO-nc:SiO_2_ and Eu^3+^:ZnO-nc:SiO_2_. Savchyn et al. [24] report a similar analysis calculating the transfer efficiency from the steady-state PL emission intensity for a silicon nanocrystals and Er^3+^ ions system. Our results on the steady-state PL intensity for the samples without and with Eu^3+^ ions have been reported in Reference [9], and we used those results to calculate the energy transfer efficiency, denoted by $E_{T}^{PL}$, in this report. The intensity of steady-state PL emission from the ZnO-nc:SiO_2_ $\left(I^{ZnO-nc:SiO_{2}}\right)$ and Eu^3+^:ZnO-nc:SiO_2_ $\left(I^{Eu^{3+}:ZnO-nc:SiO_{2}}\right)$ samples can be mathematically represented by Equations (5) and (6) [24], respectively:

(5) $$I^{ZnO-nc:SiO_{2}}\propto N^{ZnO-nc:SiO_{2}}\cdot R^{r}$$

(6) $$I^{Eu^{3+}:ZnO-nc:SiO_{2}}\propto N^{Eu^{3+}:ZnO-nc:SiO_{2}}\cdot R^{r}$$

where $N^{ZnO-nc:SiO_{2}}$ is the number of ZnO-nc per unit volume in the excited state of any emission center for the ZnO-nc:SiO_2_ sample, and $N^{Eu^{3+}:ZnO-nc:SiO_{2}}$ is the number of ZnO-nc per unit volume in the excited state of any emission center for the Eu^3+^:ZnO-nc:SiO_2_ sample. Under the steady-state condition of PL measurement, the rate equation of $N^{ZnO-nc:SiO_{2}}$ [25] can be written as follows:

(7) $$\frac{\partial N^{ZnO-nc:SiO_{2}}}{\partial t}=+G-N^{ZnO-nc:SiO_{2}}\left(R^{r}+R^{nr}\right)=0$$

where *G* is the total number of ZnO-nc generated to the excited state of an emission center per unit volume per unit time. The value of *G* depends on the intensity of the steady-state PL excitation source and the absorption cross-section of the ZnO-nc. Similarly, the rate equation of $N^{Eu^{3+}:ZnO-nc:SiO_{2}}$ [25] can be written as follows:

(8) $$\frac{\partial N^{Eu^{3+}:ZnO-nc:SiO_{2}}}{\partial t}=+G-N^{Eu^{3+}:ZnO-nc:SiO_{2}}\left(R^{r}+R^{nr}+R^{tr}\right)=0$$

Thus, from Equations (7) and (8), we can see that during the PL measurement, i.e., under a steady-state condition $\left(\frac{\partial N}{\partial t}=0\right)$, $N^{ZnO-nc:SiO_{2}}$ depends only on $R^{r}$ and $R^{nr}$ for the ZnO-nc:SiO_2_ sample and $N^{Eu^{3+}:ZnO-nc:SiO_{2}}$ depends on $R^{r},R^{nr}$, and $R^{tr}$ for the Eu^3+^:ZnO-nc:SiO_2_ sample. Here, we have assumed that the addition of Eu^3+^ ions into the SiO_2_ matrix does not affect the generation process of ZnO-nc in the excited state of any given emission center, because the intensity of the steady-state PL excitation source and the absorption cross-section of ZnO-nc are constant values in the steady-state PL measurement of the two samples. This implies that the values of *G* are the same for the samples without and with Eu^3+^ ions. Since the intensity of the PL emission is directly proportional to the number of ZnO-nc per unit volume in the excited state of an emission center, as shown in Equations (5) and (6), then using Equations (7) and (8), we have:

(9) $$\frac{I^{Eu^{3+}:ZnO-nc:SiO_{2}}}{I^{ZnO-nc:SiO_{2}}}=\frac{\frac{G}{R^{r}+R^{nr}+R^{tr}}\cdot R^{r}}{\frac{G}{R^{r}+R^{nr}}\cdot R^{r}}$$

The energy transfer efficiency from the PL steady-state measurements $\left(E_{T}^{PL}\right)$ can then be written as follows:

(10) $$\mathrm{Transfer}\;\mathrm{efficiency}\;\left(E_{T}^{PL}\right)=\frac{R^{tr}}{R^{r}+R^{nr}+R^{tr}}=1-\frac{I^{Eu^{3+}:ZnO-nc:SiO_{2}}}{I^{ZnO-nc:SiO_{2}}}$$

The values of $E_{T}^{PL}$ are shown in Figure 3, together with those of $E_{T}^{TRPL}$, for the various ZnO-nc emission centers. The figure also shows the error bar for each of the transfer efficiency values calculated using the two different methods.

We notice that the $E_{T}^{PL}$ and $E_{T}^{TRPL}$ transfer efficiencies have similar values and are well within the error bars of the two measurements methods. The $E_{T}^{PL}$ and $E_{T}^{TRPL}$ transfer efficiencies are between 0.5 and 0.7 for most of the ZnO-nc emissions, except for VӦ defect emission at 575 nm. The transfer efficiencies obtained in this work are quite similar to the energy transfer efficiencies from silicon nanocrystals to Er^3+^ ions reported by Savchyn et al. [24], calculated using similar methods. In our previous work [9], we calculated the spectral overlap integral of each of the seven ZnO-nc emissions with the Eu^3+^ excitation spectrum to obtain a measure of the energy transfer from ZnO-nc to the Eu^3+^ ions resulting in the emission at 614 nm. It has been found and reported in Reference [9] that the EE and Zn_i_ to V_Zn_ emission centers emitting at 378 and 396 nm have the highest contribution to the energy transfer from ZnO-nc to Eu^3+^ ions, while the oxygen defect emissions of ZnO-nc such as O_i_, V_o_, VȮ, and VӦ, which are centered at 417, 450, 500, and 575 nm, respectively, have low energy transfer contributions to the Eu^3+^ ions. Thus, combining the results of spectral overlap integrals from our previous work [9] with the transfer efficiencies results of this work, we can deduce that the high transfer efficiencies $E_{T}^{PL}$ and $E_{T}^{TRPL}$ of EE and Zn_i_ to V_Zn_ emission centers are mostly due to the energy transfer from ZnO-nc to Eu^3+^ ions, which results in the radiative emission from the Eu^3+^ ions at 614 nm. We also observe that for the oxygen defect emissions of ZnO-nc such as O_i_, V_o_, and VȮ, the values of the transfer efficiencies $E_{T}^{PL}$ and $E_{T}^{TRPL}$ are similar to those of EE and Zn_i_ to V_Zn_ emission centers, even though the spectral overlap integral values of oxygen defect emissions are low. This implies that the energy transfer from these oxygen defect emission centers is mostly due to the energy transfer from ZnO-nc to the additional new defects created due to the incorporation of the Eu^3+^ ions [9], which was proposed in our earlier work [9]. The formation of Eu^3+^ ion-induced defect centers is a very important result that needs to be considered when fabricating light-emitting devices which use the energy transfer process from ZnO-nc to excite RE ions. This result, together with the knowledge and understanding of ZnO-nc emission centers which contribute to the energy transfer process from ZnO-nc to Eu^3+^ ions resulting in a strong emission at 614 nm, can help in fabricating future, energy-efficient red light-emitting devices.

## 3. Materials and Methods

The samples in this study were fabricated using the low-cost sol-gel process and made into thin films of approximately 300 nm by spin-coating on a Si substrate. The sol-gel process is essentially a three-step process where, in the first step of chemical mixing the precursor, the solvent and the catalyst were added together to create the sols. Separate sols were prepared for fabricating the SiO_2_ matrix and the ZnO-nc. The SiO_2_ sol was prepared with tetraethyl orthosilicate (TEOS) as the precursor dissolved in ethanol along with de-ionized water, while the ZnO-nc sol was prepared with zinc acetate dihydrate as the precursor and diethylamine as the catalyst dissolved in ethanol. In the second step, the sols were aged for 24 h, after which Europium(III) nitrate pentahydrate was added to the SiO_2_ matrix sol to incorporate the Eu^3+^ ions. The two sols were then mixed to create samples with Zn:Si in a 1:2 ratio and spin-coated on a (100) Si substrate. Finally, the samples were soft baked for 5 m at 100 °C and subsequently annealed at 450 °C in an O_2_ environment for 60 s using the Rapid Thermal Annealing (RTP) process. The steps of fabrication are exactly same as those reported in our previous publications [9,10], and the exact same samples used in our previous study of the steady-state PL emission from ZnO-nc and Eu^3+^ ions were also used for this work [9]. The formation of ZnO-nc embedded in an SiO_2_ matrix using this sol-gel recipe has been studied in detail in our group’s earlier publication [10], wherein the TEM images of the samples were analyzed to confirm the formation of ZnO-nc.

The TRPL spectra was obtained by exciting the sample using a femtosecond amplified-pulsed laser, which was tuned to 325 nm using an optical parameter amplifier and detected using a streak camera (OptoScope SC-10, Optronis, Kehl, Baden-Württemberg, Germany) [26]. The pulse-width and the repetition rate of the excitation pulse were 100 fs and 1 KHz, respectively. The PL measurements were obtained by exciting the samples at 325 nm using a spectrofluorometer (SPEX Fluorolog-3 Model FL3-11, Horiba, Edison, NJ, USA).

## 4. Conclusions

In this work we measured the TRPL spectra of ZnO-nc:SiO_2_ and Eu^3+^:ZnO-nc:SiO_2_ samples to study the contribution of energy transfer to the Eu^3+^ ions from the various emission centers of ZnO-nc. The TRPL spectra of the two samples were analyzed by calculating the decay time and the stretching exponential coefficient values of the different ZnO-nc emissions centers at 360, 378, 396 and 417 nm using the stretched exponential decay function. The decay time showed that samples with Eu^3+^ ions experience faster decay because of the additional components, namely the energy transfer from ZnO-nc to the Eu^3+^ ions, which results in the radiative emission from the Eu^3+^ ions at 614 nm, and the energy transfer to the additional new defects created due to the incorporation of the Eu^3+^ ions, contributing to the decay rate. Furthermore, the transfer efficiencies of the ZnO-nc emissions calculated from the decay time are consistent with the transfer efficiencies calculated using the PL emission intensity data. These results show that the high transfer efficiencies of EE and Zn_i_ to V_Zn_ emission centers emitting at 378 and 396 nm is mostly due to the energy transfer from ZnO-nc to Eu^3+^ ions, resulting in an emission at 614 nm, while the energy transfer from the oxygen defect emissions at 417, 450, 500 and 575 nm is most probably due to the energy transfer from ZnO-nc to the defects created due to the incorporation of the Eu^3+^ ions in the Eu^3+^:ZnO-nc:SiO_2_ samples, as proposed in our previous work. Moreover, the stretching exponential coefficient values, which are a measure of interaction between two or more identical ZnO-nc emission centers, show that the 360-nm band edge emission (QC) experiences lesser interactions compared to the EE, Zn_i_ to V_Zn_, and O_i_ emissions centered at 378, 396, and 417 nm, respectively. These are important results which will help in the fabrication of future efficient light sources based on the energy transfer process from semiconductor nanocrystals.

## Figures and Tables

**Table 1:** The decay time (*τ*) and stretching exponential coefficient (*β*) values for the various ZnO-nc emission centers obtained from the time-resolved photoluminescence (TRPL) spectra of the samples with and without Eu^3+^ ions (Eu^3+^:ZnO-nc:SiO_2_ and ZnO-nc:SiO_2_ samples, respectively).

Sample	Emission Wavelength	τ (ps)	β
ZnO-nc:SiO_2_	360 nm	215 ± 8	0.65 ± 0.02
Eu^3+^:ZnO-nc:SiO_2_	(QC)	102 ± 7	0.69 ± 0.02
ZnO-nc:SiO_2_	378 nm	213 ± 4	0.62 ± 0.01
Eu^3+^:ZnO-nc:SiO_2_	(EE)	84 ± 4	0.62 ± 0.01
ZnO-nc:SiO_2_	396 nm	264 ± 6	0.61 ± 0.01
Eu^3+^:ZnO-nc:SiO_2_	(Zn_i_ to V_Zn_)	95 ± 5	0.61 ± 0.01
ZnO-nc:SiO_2_	417 nm	356 ± 15	0.62 ± 0.02
Eu^3+^:ZnO-nc:SiO_2_	(O_i_)	125 ± 10	0.62 ± 0.02
